# The COVID-19 response must integrate people living with HIV needs in Sub-Saharan Africa: the case of Mali

**DOI:** 10.1186/s41182-020-00228-5

**Published:** 2020-06-03

**Authors:** Luis Sagaon-Teyssier, Adam Yattassaye, Michel Bourrelly, Bintou Dembélé Keïta, Bruno Spire

**Affiliations:** 1 Aix Marseille Univ, INSERM, IRD, SESSTIM, Sciences Economiques & Sociales de la Santé & Traitement de l’Information Médicale, Marseille, France; 2 ARCAD Santé PLUS, Bamako, Mali

**Keywords:** COVID-19 prevention, HIV continuum of care, Public health, Mali, NGO implication

## Abstract

The first COVID-19 cases in Mali were reported almost 1 month after the first case in the African continent. However, the outbreak continues to spread faster there than in other countries which, along with Mali, successfully tackled the 2014 Ebola outbreak in Africa. Given this context, specific actions targeting people living with HIV (PLWH) are needed to reinforce prevention. Community-based involvement is crucial to ensure continuity of care and treatment for PLWH. Furthermore, the health of frontline healthcare workers must take priority in any actions taken. The long-established trustful relationship between NGOs and PLWH in Mali is indispensable to disseminate key messages about COVID-19.

To the Editor,

Mali announced its first two cases of coronavirus disease 2019 (COVID-19) on March 25, 2020, almost 1 month after Algeria (first reported case in the African continent) and Nigeria (first reported case in Sub-Saharan Africa). The Ministry of Health and Social Affairs communiqués reported 631 cases (331 active, 32 deaths, 261 recoveries, and 7 cases transferred outside the country) and 2016 persons under observation as of May 6 [1].

While the evolution of the outbreak in Mali is not yet clear, it continues to spread faster there than in Algeria and Nigeria (Fig. 1a) and faster than in the Democratic Republic of Congo (DRC) and Senegal, which, together with Mali and Nigeria, were the 4 countries which successfully tackled the 2014 Ebola outbreak in Africa (Fig. 1b). This is despite the fact that Malian authorities were more proactive in implementing a response than their counterparts in these other countries when the WHO declared COVID-19 to be an international public health emergency. Specifically, systematic body temperature screening was implemented on March 12—2 weeks before the first announced cases— at Modibo Keita International Airport, one of Mali’s main entry points for imported disease cases. Nevertheless, the virus’s continued progression throughout the continent suggested that this measure was inadequate [2, 3]. Accordingly, the authorities decided to halt all air traffic. Total interruption of commercial flights took effect 5 days *before* the first cases, much earlier than DRC, Senegal, and Nigeria (11, 19, and 25 days *after* their first cases, respectively) (Fig. 1b). Other proactive decisions, including measures to ban mass gatherings (March 23, 2 days before) and a curfew (March 26, 1 day after), distinguish Mali’s response from those of these other countries (Fig. 1b).

**Fig. 1:**
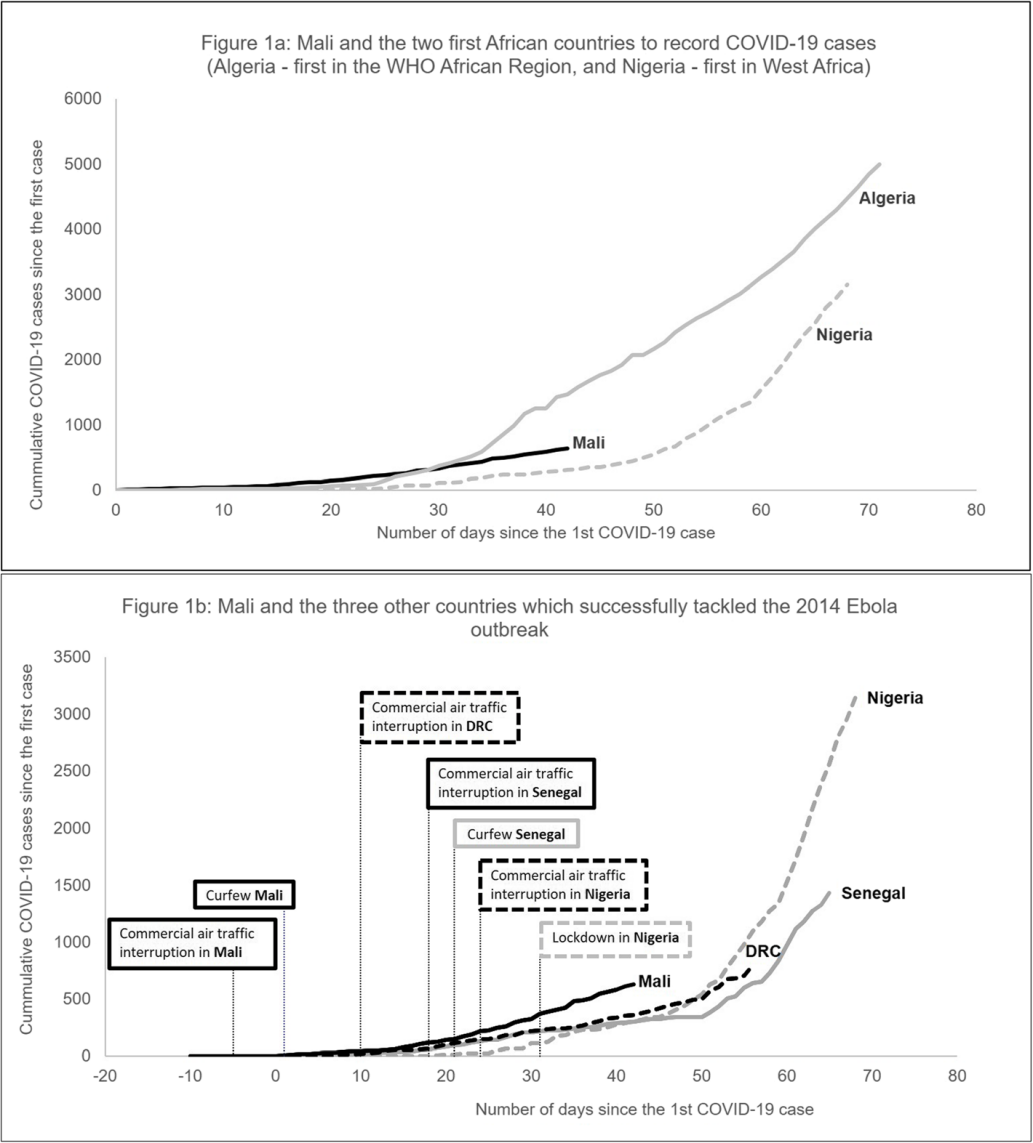
Cumulated COVID-19 cases by the number of days since the first recorded case. Source: authors’ construction using data from the WHO Africa dashboard, https://www.afro.who.int/health-topics/coronavirus-covid-19 (data updated May 6, 2020). For Mali, data on daily COVID-19 cases were obtained from the official communiqués from the Ministry of Health and Social Affairs (data updated May 6, 2020) http://www.sante.gov.ml/index.php/actualites. *In Mali, all commercial air traffic was interrupted from/to the most affected countries 5 days before (i.e., March 20, 2020) the first reported COVID-19 cases. In Senegal and RDC, all commercial air traffic was interrupted on March 20, 2020: https://www.bbc.com/afrique/region-51959820 and https://www.bbc.com/afrique/region-51959819. In Nigeria, all air traffic was interrupted on March 23, 2020: http://covid19.ncdc.gov.ng/resource/DOC210320-21032020144614.pdf. Curfew in Mali was implemented on March 26, 2020 (Décret n°2020-0170/P-RM): http://malijet.com/communiques-de-presse/241030-couvre_feu_mali_invite_forces_ordre_.html. The curfew in Senegal was implemented on March 23, 2020: http://www.sante.gouv.sn/sites/default/files/Discours%20Pr%C3%A9sident%20de%20la%20R%C3%A9publique%20%20%C3%A9tat%20d%27urgence%20COVID-19.pdf. The lockdown in Nigeria’ main cities was implemented on March 30, 2020: https://www.aljazeera.com/news/2020/03/nigeria-announces-lockdown-major-cities-curb-coronavirus-200330095100706.html

During the 2014 Ebola outbreak, the lag between the first cases in Africa and the outbreak in Mali and the abovementioned countries helped them to prepare [4]. Furthermore, international aid arrived quickly. In contrast, aid for COVID-19 has been slower to arrive, probably because of the virus’s pandemic status. Consequently, Mali’s current challenge is to implement an effective response despite few healthcare resources.

Mali, just like most Sub-Saharan African countries, faces major healthcare challenges in the fight against COVID-19 [5]. At the beginning of the outbreak, the country had only 49 hospital beds available. Personal protective equipment (PPE), hospital equipment (respirators, infrared thermometers, etc.), and laboratory reagents are all in short supply [6]. Given this context, actions targeting cultural and social dimensions of vulnerable populations—including the homeless [7] and individuals with endemic [8] and chronic diseases, especially people living with HIV (PLWH) [9]—are needed to reinforce COVID-19 prevention. The pandemic is already impacting PLWH continuity of care and treatment [10]. Furthermore, the physical and mental health of healthcare workers (HW) must take priority in any response taken [11, 12]. As for continued HIV prevention and care during the current crisis, community-based involvement is crucial to ensure that vulnerable populations are reached [13]. On April 1, 2020, ARCAD-Santé PLUS, the main Malian NGO working on improving access to healthcare for PLWH and vulnerable populations since 1994, launched the CovidPrev project in its 22 structures (18 healthcare sites and 4 sexual healthcare centers) operating in 6 of the country’s 10 administrative regions. CovidPrev’s main objective is to reduce the risk of the virus propagating in HW, in the 28878 PLWH frequenting the NGO’s structures, and in other vulnerable populations (people over 55, people with co-morbidities, sex-workers, men who have sex with men, and injecting drug users). More specifically, it aims to (i) implement effective measures to prevent COVID-19, (ii) guarantee the HIV care continuum and social assistance (moral and material) for these vulnerable populations, and (iii) manage medical emergencies (HIV screening, new enrolments, medical consultations, etc.). The specific actions for each of the aims (i, ii, iii) are as follows:

i) Distribute PPE to HW and vulnerable populations, create a centralized information platform for staff in the NGO’s 22 structures, reorganize healthcare services to reduce patient flow and waiting times, and distribute COVID-19 prevention messages to vulnerable populations through web/mobile platforms and a free hotline
ii) Test and treat for HIV, deliver 6-month HIV antiretroviral treatment (ARV) provisions to space out appointments for vulnerable service users, deliver ARV to mobility-reduced persons, and distribute food kits to those most in need
iii) Provide medical permanence for medical emergencies and referral to hospitals, continue new enrollments in structures, and deliver ARV to newly diagnosed PLWH

The actions listed above must be accompanied by research. More specifically, prospective research in the field of social sciences and public health is vital for NGOs in Africa and elsewhere to further knowledge about the impact of this new virus on HW and vulnerable populations’ living/working conditions. This is especially true for Mali, a country with a fragile healthcare system, where economic hardship constitutes one of the main barriers to people being able to follow government regulations aimed at preventing the spread of COVID-19.

The long-established trustful relationship between ARCAD-Santé PLUS and users of its HIV/AIDS prevention and care services is indispensable to disseminate key messages to protect vulnerable populations in Mali against COVID-19.

## Data Availability

All the data used in this letter are drawn from the references provided.

## Abbreviations

AIDS: Acquired immune deficiency syndrome
ARV: Antiretroviral treatment
COVID-19: Coronavirus disease 2019
DRC: Democratic Republic of Congo
HIV: Human immunodeficiency virus
HW: Healthcare workers
NGO: Non-governmental organization
PLWH: People living with HIV
PPE: Personal protective equipment
